# 

**Published:** 1915-01

**Authors:** 


### SUBJECT INDEX TO VOLUME LXIX

EVENT AND COMMENT.

American Dental Hospital, 351.
American Dental Service to Allied Armies, 504.
American Institute of Dental Teachers, 53.
Conductive Anesthesia, 43.
Contents of New Dental Curriculum, 102.
Copper Cements, 151.
Defending the Profession from Malpractice Suits, 404.
Director Eastman Dispensary, 503.
Eastman Dental Benefaction, 351.
Four Year Course, 51.
Future Preventive Dentistry, 401.
Greatest Problem of the Day in Dentistry, 152.
Green V. Black, 451.
New Registration Law, 201.
Ohio State Dental Society, 1.
Panama-Pacific Dental Congress, 301, 503.
Professor Cassidy Quits Teaching, 502.
Shall the Added Year be a Real Advance? 251.
Silver Loving Cup for Donors of Forsythe Institute, 253.
Sterilizing the Operative Field, 43.
The Future of Dental Education, 3.
The Miller Memorial, 505.
University Schools Decide on a Curriculum, 301.
What Have We Done? 551.
What of 1915? 3.
What Shall We Do with the Dental Pulp? 202.

FORMAL PAPERS.

A 1915 Code of Dental Ethics, H. E. Dennett, 34.
All Over and Doing Well, Mary C. Raymond, 70.
A Method of Exactly Locating Gilmore Attachments, E. P. Grant, 11.
Anesthesia, H. D. Henderson and N. F. Jones, 306.
A Novelette, R. Ottolengui, 105.
Asepsis and Antisepsis in Dentistry, A. Kuehn and J. A. Kimmel, 310.
A Tribute to Dr. Stockwell. By Dr. James McManus, 515.
Cause and Cure of Pyorrhoea by Emetin, 6.
Cause of Dental Diseases and Present Day Practices, Otto E. Inglis, 374.
Constitutional Infection Due to Abscess and Pyorrhoea, L. W. Doxtater, 470.
Copper Cement Chemistry, W. S. Medell, 172.
Dosage in Vaccine Therapy, G. H. Sherman, M. D., 214.
Endamebic Pyorrhoea and its Complications, J. S. Evans and W. S. Middleton, 55.
Endowed Dental Dispensary for Rochester, N. Y., 355.
Etiologic Relations of Facial Infections to Systemic Disease, N. W. Jones, M. D., 12.
Fallacies and Facts, H. E. Bliler, 407.
Fees for Dentures Made on a Commercial and Professional Basis, W. E. Bailey, C. P. Brown, 257.
First Report American Dental Ambulance. By Dr. George B. Hayes, 506.
Four Years' Curriculum, E. C. Kirk, 270.
Harrison Anti-Narcotic Bill, 64.
How Should Dentists Advise People to Clean Their Teeth? B. Feldman, 573.

Importance of Sound Temporary Teeth. By J. L. Young, 526.
Indirect Inlay Method, H. W. Gillett, 284.
Is Boric Acid Good for Babies? Dr. R. A. Albray, 522.
Medical School Attendance, 72.
Memorial to Dr. G. V. Black, 528.
Mouth Toilet, R. C. Ling, C. R. Mull, 313.
New Building of Evans Institute, 121.
New Standards and Old, 281.
Oral Sepsis and Children's Diseases, 279.
Pain, W. L. French, R. L. Felton, 267.
Paris Dental Hospital, 353.
Physical Diagnosis and its Importance to Dentists, W. C. Collins, I. N. Gallagher, 264.
Plaster, Vulcanite and Esthetics, Geo. H. Wilson, 112.
Porcelain Facings. By John H. Penhale and Harold E. Rice, 517.
Practitioner's Part in Oral Health Propaganda, Harold Clark, 132.
Preparation of the Mouth for Artificial Tooth, R. Brown and C. P. Bower, 254.
Prevention of Dental Caries and Pyorrhoea, A. C. Lucas and C. M. Mate, 275.
Prevention the Technique of the Future, C. H. Gerrish, 417.
Professional Ideals and Practical Patients, E. Harrison, L. D. S., 555.
Protest Against Extracting Pulpless Teeth, C. N. Johnson, 217.
Review of Articles on Root Canal Treatment, R. Ottolengui, 410.
Section on Stomatology as a Factor in the Evolution of Medical and Dental Science. G. V. I. Brown, 19.
Some Fallacies in the Treatment of Phenol Poisoning, 388.
Special Material Used for Casts in Vulcanite Work, J. H. Prothero, 203.
Special Richmond and Goslee Crowns, A. J. Nishon and E. J. Reynolds, 321.
Spread of Infectious Diseases by Schools, 429.
Studies in Mouth Pathology and Toxin Absorption, by J. Endelman and J. D. McCoy, 454.
Technique of Oral Prophylaxis, Arthur Day, 211.
The Germicidal Efficiency of Cements, Paul Poetschke, 153.
The Greatest Problem of the Day in Dentistry, R. Ottolengui, 189.
The House; its Unique Problems in Hygiene, 426.
The Human Mouth; its Care from Birth to the Completion of First Dentition, J. G. O'Neil, 581.
The Newer Ideas on Ventilation, 430.
The Technique and Balancing of Local Anesthetics, J. Novitzky, 181.
The Tooth; Does it Help or Hinder Oral Cleanliness, 566.
The Trend of Dentistry, A. E. Webster, 119.
Tuberculosis and How to Combat it, 431.
Tuberculosis; its Development in Childhood, T. Frazer, 422.
Twilight Sleep in Dentistry, M. L. Rudolph, 180.
Useful Adjunct to Emetin Treatment of Pyorrhoea, H. A. White, 405.
Who is an Orthodontist? Martin Dewey, 286.
Why We Should Buy Goods Made in U. S. A., I. W. Lambert, 75.
Working Silicate Cements, Chas. E. Jones, 361.

BIBLIOGRAPHY.

American Illustrated Dictionary, 491.
Appleton's Medical Dictionary, 593.
Bass and Johns' Alveolo Dental Pyorrhoea, 305.

Black, Pathology, 303.
Brophy's Oral Surgery, 591.
Burchard, Inglis Pathology, 304.
Johnson's Operative Dentistry, 448.
Noyes' Ethics and Jurisprudence, 219.
Practical Oral Hygiene, Prophylaxis and Pyorrhoea, 448.
Principles and Technique of Crowns and Bridges, 218.

OBITUARY.

Chauncey S. Bigelow, 46.
Green V. Black, 499.
Charles R. Butler, 45.
Louis Jack, 248.
Ralph E. Luther, 241.
H. B. McFadden, 242.
Wm. P. Morgan, 44.
W. X. Sudduth, 242.
James Truman, 47-80, 246.
Faneuil D. Weisse, 332.
J. B. Wilmott, 337.

INDENTS.

A Full Practice, 488.
A Way to Keep Oil of Cajuput, 296.
Accidents in Operating, 486.
Acid Burns, 531.
Acute Ulcerous Gingivitis, 538.
Adhesion and Cohesion of Cements, 138.
Adjustment of Clasps, 489.
Alcohol as a Germicide, 83.
Alcohol Injection in Fifth Nerve, 338.
Aluminum and Vulcanite Plates, 230.
Alveolar Abscess and Systemic Infection, 233.
Amalgam and Gold Restorations, 222.
Amalgam as a Filling Material, 138.
Amalgam Restorations, 224.
Ameba in Pyorrhoea, 436-441.
Amoeba, 333.
American Dental Hospital in Paris, 84.
An Unusual Case Treated Unsuccessfully with Emetin, 438.
Apical Infection, 332.
Application of Radium in Dentistry, 292.
Applied Science and Dentistry, 290.
Army Dental Surgeons, 236.
Arthretis Deformans, 141.
Aseptic Canal Procedures, 332.
Aseptic Pericementitis, 331.
Autogenous Vaccines, 137.
Bone Development in Infra-Occlusion, 295.
British Soldiers' Teeth, 337.
Buckley's Desensitizing Paste, 235, 328.
Care of Soldier's Jaw Wounds, 435.
Care of Teeth in the Armies, 82.
Cast Aluminum Dentures, 193.
Casting Cusps for Banded Crowns, 88.
Cause of Gingivitis, 437.
Cause of Pyorrhoea, 225.
Cause of Pyorrhoea Pockets, 489.
Cause of Dental Caries, 477.
Caution in Diagnosis, 226.
Coating for Plaster Casts, 532.
Cold Air as an Obtundent, 335.
Compensating Shrinkage in Inlays, 532.
Cutting Sensitive Dentine, 536.
Danger in Anesthesia, 533.
Dangers of Local Anesthesia, 483.
Deaths from Anesthesia in England, 400.
Dental Commandments, 80.
Dental Foci of Infection, 139.
Dental Licentiate Inadequate, 225.
Dental Toilet, 42.
Desensitizing Paste in Crowning Teeth, 195.
Doing Away with Pumice, 531.
Does Pyorrhoea Cause Iriti? 87.
Emetin, 223, 232, 237.
Emetin a Double Specific, 38.
Endamoeba in Pyorrhoea, 440.
Endamoeba and Emetin, 475.
Errors in Diagnosis, 137.

Essentials for Local Anesthesia, 334.
Extracting Badly Broken Molars, 393.
Filling Root Canals, 335.
Filling Root Canals with Paste, 333.
Filling Small Root Canals, 334.
First Aid in Artificial Respiration, 85.
Forsythe Dental Infirmary, 37, 83, 441.
Forsythe Infirmary Saving the Teeth of Boston's Children, 289.
Gold Fillings versus Gold Inlays, 194.
Good Soap, 88.
Hand Lotion for Chapped Hands, 79.
Hot Drinks Cause Gingivitis, 435.
How to Have Good Health, 42.
Ideation Under Anesthesia, 446.
Improved Implantation, 230.
Improving Color of Pink Rubber, 223.
Indictment of the Dentist, 446.
Indirect Method of Inlay Making, 393.
Infective Foci in the Mouth, 140.
Infected Third Molar Sockets, 488.
Inlays by Indirect Method, 476.
Investing to Cast a Cusp, 88.
Iodine Stains, 88.
Is the Entamoeba Always Present? 438.
Jacket Crown, The, 478.
Lack of Legalized Dentists in Europe, 398.
Local Treatment of Pyorrhoea, 145.
Locating Canal Openings, 42.
Medical Prisoners Exchanged, 81.
Melting Aluminum, 340.
Mixing Amalgam, 142.
Modeling Compound Impressions, 238.
More Time in Using Mouth Washes, 535.
New Jersey Societies, 290.
New York's Great Need, 535.
Novocaine and the War, 81.
Obtunding Dentine, 537.
Origin of Alveolar Infection, 227.
Our Aim—A Better Individual Dentist, 37.
Pain Caused by Dentures, 196.
Partial Removable Dentures, 442.
Peridental Anesthesia, 335.
Points in Pyorrhoea Treatment, 534.
Poisonous Mushrooms, 41.
Preliminary Treatment of Peridentitis, 434.
Preventing Slipping of Cord, 297.
Proportions of Tooth Extractions, 82.
Prophylaxis Treatment of Roots, 442.
Protecting Gold Work from Amalgamation, 222.
Pyorrhoea a Cause of Serumal Tartar, 237.
Pyorrhoea Begins in the Gums, 439.
Pyorrhoea Pockets, 443.
Recovering Mercury, 231.
Reconstructing Gold Inlay Margins, 229.
Removing Tin from Plates, 488.
Removing Amalgam from Fingers, 240.
Removable and Saddle Bridges, 198.
Removing Ink Stains, 240.
Removing Plaster from Vulcanite Plates, 435.
Removing Rust from Hand-piece, 229.
Removing Stains from Necks of Teeth, 393.
Responsibility for Patient's Health, 532.
Restricting Flow of Solder, 532.
Results of Implantation, 84.
Retention and Burnishing Inlay Matrices, 340.
Right to Recover for Dental Services, 490.
Sanitary Bridge Work, 144.
Saving Tooth Tissue, 223.
Scaling Pyorrhoea Pockets, 439.
Silicate Fillings, 395.
Spence Metal Models, 482.

Sterilizing Canal Instruments, 531.
Sterilizing the Tooth Brush, 391.
Sterilizing Instruments, 240.
Still Another Palagra Theory, 40.
Supplying Missing Teeth, 391.
Swaged Cast Plates, 145.
Teeth a Menace to Health, 143.
Temporary Stopping, 222.
The Dentist, 447.
The Doctor Title for German Dentists, 83.
The Tooth Pulp, 480.
To Sterilize Instruments, 299.
Tooth Brushing Hour in School, 333.
Treatment of Interstitial Gingivitis, 238.
Treatment of Sensitive Cavities, 197.
Unsanitary Tooth Brush Substitute, 397.
Value of Normal Occlusion, 296.
Vital Pulps, 481.
What Can an Unsuccessful Graduate Dentist Do? 221.
What Next in Treatment of Pyorrhoea? 444.

BIOGRAPHIC.

Albray, R. A., 522.
Angel, A. C., 199.
Ash, C. F., 223.
Atkinson, Wm. M., 240.
Bailey, W. E., 257.
Baker, D. W., 398.
Bass and Johns, 39.
Bayles, D. D., 240.
Beaumont, W. M., 87.
Best, E. S., 333.
Black, A. D., 30.
Black, G. V., 528.
Bliler, H. E., 407.
Bliven, F., 537.
Boak, S. D., 237.
Bonwill, Dr., 443.
Bower, Chas. P., 254.
Brophy, T. W., 197.
Brown, R., 254.
Brown, C. P., 257.
Brown, C. E., 198.
Brown, G. V. I., 19, 34.
Buckley, J. P., 331, 333, 335.
Bunting, R. W., 337.
Burkhart, H. J., 503.
Cassidy, J. S., 501.
Chance, A. W., 17.
Clark, Harold, 132.
Collins, W. C., 264.
Copon, Dr., 481.
Couzett, J. V., 195.
Cummer, W. E., 145.
Dannehower, A. F., 222.
Day, Arthur, 211.
De Ford, Dr., 446.
Dennett, H. E., 34.
Dewey, M., 286.
Dilger, F. S., 240, 296.
Doxtater, L. W., 470.
Elliott, Chas. W., 292.
Endelman, Julio, 454.
Evans, J. S., 55.
Evans, Thomas W., 130.
Feldman, B., 573.
Felton, R. L., 267.
Fisher, W. C., 33.
Frazer, T., 422.
French, G. H., 41.
French, W. L., 267.
Gallagher, J. N., 264.
Gerrish, C. H., 417.
Gillett, H. W., 222, 223, 284.
Gilmer, Thos. L., 137, 437.
Goslee, H. J., 340.
Grant, S. P., 11.
Hall, John S., 216.
Harrison, E., 555.
Harrison, J. H., 196.
Harper, W. E., 143.
Hartzell, L. B., 437, 438, 439, 441, 442.
Hayes, Geo. B., 504, 506.
Head, Joseph, 28.
Henderson, H. D., 306.
Hopewell, Smith, 478.
Hopkins, J. C., 532.
Hovestadt, J. F., 218.
Howe, Percy R., 147.
Inglis, Otto E., 374.
Johnson, C. N., 217, 534, 538.
Jones, Chas. E., 361.

Jones, N. F., 306.
Jones, N. W., 12.
Kauffer, H. J., 84.
Kehoe, F. P., 445.
Kells, C. E., 332.
Kimmel, J. A., 310.
Kirk, E. C., 131, 270.
Kramer, F. B., 235.
Kuehn, A., 310.
Lambert, J. W., 75.
Landers, T. A., 392.
Leach, T. A., 477.
Lee, A. P., 531.
Lemke, A. H., 82.
Lieman, R. C.. 476.
Linscott, A. F., 232.
Loomis, A. G., 8.
Lucas, C. D., 233.
Lucas, S. C., 275.
Lyng, L. C., 313.
Lyons, C. J., 335.
MacNeil, W. H., 227.
Mason, Walt, 447.
Mayer, D., 231.
Mayo, C. H., 140.
McCoy, J. D., 454.
McManus, Dr., 489.
McManus, James, 515.
Medell, W. S., 172.
Middleton, W. S., 55.
Miller, W. D., 505.
Morgan, H. W., 226.
Morehead, F. B., 29.
Mote, C. M., 275.
Mull, C. R., 313.
Nishon, A. J., 321.
Novitzky, J., 181.
Novilsky, J., 538.
Noyes, E., 219.
Oakman, C. H., 38.
O'Neil, J. G., 581.
Ottolengui, R., 105, 189, 410, 480.
Parker, R. W., 399.
Pecan, P. J., 240.
Penhale, John H., 517.
Perdue, E. M., 41.
Pike, Jay N., 229.
Poetschke, Paul, 153.
Potts, H. A., 486.
Prinz, H., 295, 334.
Prothero, J. H., 203.
Raper, H. R., 441.
Raymond, Mary C., 70.
Reynolds, E. J., 321.
Rice, Harold E., 517.
Rice, M. W., 395.
Roach, F. E., 391.
Rosenow, E. C., 138.
Rudolph, M. L., 180.
Salisbury, A. G.. 89.
Schwartz, F. F., 393.
Shaddock, F. J., 537.
Sherman, G. H., 214.
Skinner, F. H., 341.
Smedley, V. C., 297.
Smith, B. Holly, 540.
Smith, D. D., 143.
Smith, W. C., 393.
Starr, A. R., 236.
Stillman, P. R., 535.
Stockwell, C. T., 515.
Strong, L. W., 145.
Talbott, E., 31, 237, 238, 333, 444.
Tracy, W. D., 480, 532.
Tyrell, E. J., 435.
Ulrich, H. L., 225.
Van Woert, Dr., 43.
Watkins, L. G., 483.
Webster, A. E., 119, 224, 487.
Wheeler, H. L., 225.
White, H. A., 405.
Wilson, Geo. H., 112.
Wunsche, E., 194.
Young, J. Lowe, 526.

ANNOUNCEMENTS

American Institute of Dental Teachers, 148, 450.
Army Dental Examinations, 148.
Arizona Dental Society, 94.
B. Holly Smith's Removal, 540.
California Registration, 492.
Chattanooga Dental Society, 50
Delayed Publication, 94.
Dental Bur, 94.
Federation Dentaire, 49.
Forsythe Post Graduate School, 547.
Illinois Dental Society, 147.
Indiana Dental Association, 92. 200.

International Journal of Orthodontia, 93.
N. S. Jenkins Banqueted, 338.
Kansas Dental Association, 147.
Kentucky Dental Association, 147, 200, 243.
Michigan Examinations, 200, 243, 450.
Michigan Dental Society, 148.
Missouri Dental Association, 148.
National Mouth Hygiene Association, 92, 541.
National Association Dental Faculties, 540.
Northern Ohio Dental Association, 20.
Officers National Dental Association, 540.
Officers Ohio Dental Society, 150.
Ohio State Society Program, 548.
Oklahoma Dental Association, 92.
Panama-Pacific Congress, 94, 298, 342.
Panama-Pacific Dental Congress, 540.
Patterson and Brady Banqueted, 337.
Protective Association, 243.
Relief for Wounded Soldiers, 149.
State Board of Examiners, 550.
State Board Meeting, 245.
Tennessee Dental Association, 147, 200, 242.
Texas Dental Association, 93.
United States Navy Service, 492.

### ANNOUNCEMENTS.

### Federation Dentaire Internationale at the Panama-Pacific International Exposition, San Francisco, 1915.

Another assurance that the European war will not harm the Panama-Pacific International Exposition came today to James A. Barr, director of congresses and conventions for the exposition. The Federation Dentaire Internationale, the highest authority in dental matters, has chosen San Francisco as the 1915 meeting place from August 30 to September 9th.

The international character of this great organization is shown by the fact that the honorary president, Dr. W. B. Patterson, is of London; Dr. Truman H. Brophy, president, is of Chicago; the vice-presidents are of Paris, Edinburgh, Vienna, Buenos Aires, Frankfort-on-Main, Naples and Strassburg; the secretary-general is from Madrid; and the assistant secretaries are of Brussels, The Hague, Paris and Madrid.

This organization has charge of the organization of all International Dental Congresses and is the clearing house of the most important dental questions. It is made up of the individual members of the profession, and according to Dr. Flood, who has been active in bringing the organization to San Francisco, it means a great attendance although there are no regular delegates.

The entertainment of its members will be in the hands of the organizations of all the Pacific coast states.

Another great dental organization, the Delta Sigma Delta fraternity, strong in the United States, Central America, and South America, yesterday notified the exposition that it would meet during the same period as the International Federation. This is the third dental fraternity announcing its intention of meeting here the coming year and makes a clean sweep of every dental organization of the United States except the state societies, many of which also will be here.

The attendance of the supreme chapter meeting of Delta Sigma Delta is estimated by Dr. Flood at from 3,000 to 10,000 and the International Federation sessions may draw many more than this number.

Dr. Burton L. Thorpe, of St. Louis, is secretary of the International Federation and head of the Delta Sigma Delta fraternity. He has been influential in bringing both meetings to San Francisco.

---

CHATTANOOGA DENTAL SOCIETY.—The annual business meeting of the Chattanooga (Tennessee) Dental Society was held recently, during which the following officers were elected: President, Dr. Wm. F. Stone; Vice-President, Dr. N. C. Hunt; Secretary, Dr. I. R. Stone; Treasurer, Dr. George W. Wagner.

A paper read by Dr. R. S. Henry on vaccine therapy in oral infections provoked much interesting discussion.

The society is planning to give semi-annual banquets to which dentists of national reputation will be invited to deliver scientific addresses. This organization, like all others in the "Dyname of Dixie," is characterized by the qualities of life and progressiveness, the latter being demonstrated by the members' desire to keep in touch with the latest thought and discoveries in connection with their profession, by hearing the principal exponents thereof in their own midst. Incidentally, they anticipate little trouble in getting the men they desire to hear, to visit Chattanooga, which is a very attractive place to visit on account of its historic and scenic wonders, such as Lookout Mountain, Chickamauga Park, Missionary Ridge, Signal Mountain, beautifully winding and picturesque Tennessee River—every one of them in some way identified with stirring events of the Civil War.

I. R. STONE, *Secretary.*

